# Genetically Modified Rabies Virus Vector-Based Rift Valley Fever Virus Vaccine is Safe and Induces Efficacious Immune Responses in Mice

**DOI:** 10.3390/v11100919

**Published:** 2019-10-08

**Authors:** Shengnan Zhang, Meng Hao, Na Feng, Hongli Jin, Feihu Yan, Hang Chi, Hualei Wang, Qiuxue Han, Jianzhong Wang, Gary Wong, Bo Liu, Jun Wu, Yuhai Bi, Tiecheng Wang, Weiyang Sun, Yuwei Gao, Songtao Yang, Yongkun Zhao, Xianzhu Xia

**Affiliations:** 1 College of Wildlife and Protected Areas, Northeast Forestry University, Harbin 150040, China; Zhang_Shengnan1992@163.com; 2 Changchun Veterinary Research Institute, Chinese Academy of Agricultural Sciences, Changchun 130000, Chinajin8616771@163.com (H.J.); yanfh1990@gmail.com (F.Y.); ch_amms@163.com (H.C.); whl831125@163.com (H.W.); hanqiuxue93@126.com (Q.H.); wgcha@163.com (T.W.); sunweiyang1987@163.com (W.S.); gaoyuwei@gmail.com (Y.G.); 3 Comparative Medicine Center, Peking Union Medical College (PUMC) and Institute of Laboratory Animal Sciences, Chinese Academy of Medical Sciences (CAMS), Beijing 100021, China; Haorm_23@126.com; 4 Key Laboratory of Jilin Province for Zoonosis Prevention and Control, Changchun 130000, China; 5 College of Veterinary Medicine, Jilin University, Changchun 130062, China; 6 Animal Science and Technology College, Jilin Agricultural University, Changchun 130118, China; wjzd2005@163.com; 7 Institute Pasteur of Shanghai, Chinese Academy of Science, Shanghai 20031, China; garyckwong@hotmail.com; 8 Beijing Institute of Biotechnology, Beijing 100071, China; liubo7095173@163.com (B.L.); junwu1969@163.com (J.W.); 9 CAS Key Laboratory of Pathogenic Microbiology and Immunology, Institute of Microbiology, Chinese Academy of Sciences, Beijing 100101, China; beeyh@im.ac.cn

**Keywords:** Rift Valley fever virus, rabies virus, inactivated vaccine, RVFV-specific IgG antibodies, adjuvant

## Abstract

Rift Valley fever virus (RVFV), which causes Rift Valley fever (RVF), is a mosquito-borne zoonotic pathogen that causes serious morbidity and mortality in livestock and humans. RVF is a World Health Organization (WHO) priority disease and, together with rabies, is a major health burden in Africa. Here, we present the development and characterization of an inactivated recombinant RVFV and rabies virus (RABV) vaccine candidate (rSRV9-eGn). Immunization with rSRV9-eGn stimulated the production of RVFV-specific IgG antibodies and induced humoral and cellular immunity in mice but did not induce the production of neutralizing antibodies. IgG1 and IgG2a were the main isotypes observed by IgG subtype detection, and IgG3 antibodies were not detected. The ratios of IgG1/IgG2a > 1 indicated a Type 2 humoral immune response. An effective vaccine is intended to establish a long-lived population of memory T cells, and mice generated memory cells among the proliferating T cell population after immunization with rSRV9-eGn, with effector memory T cells (T_EM_) as the major population. Due to the lack of prophylactic treatment experiments, it is impossible to predict whether this vaccine can protect animals from RVFV infection with only high titres of anti-RVFV IgG antibodies and no neutralizing antibodies induced, and thus, protection confirmation needs further verification. However, this RVFV vaccine designed with RABV as the vector provides ideas for the development of vaccines that prevent RVFV and RABV infections.

## 1. Introduction

Rift Valley fever (RVF) is an important mosquito-borne viral zoonosis that threatens the health of a wide range of animals, particularly ruminants and humans. It is caused by Rift Valley fever virus (RVFV), a biosafety level 3 (BSL-3) pathogen that can be transmitted by more than 30 mosquito species [1,2,3,4,5]. RVFV belongs to the genus Phlebovirus in the family Phenuiviridae (order of Bunyavirales) [6]; contains a tripartite, negative single-stranded RNA genome; and is composed of three segments [7]: a large (L) segment, a medium (M) segment and a small (S) segment. The L segment encodes the viral RNA polymerase. The M segment encodes a glycoprotein, which can be cleaved into the glycoproteins Gn and Gc by cellular proteases during translation, as well as two non-structural proteins, a 78-kDa protein and a 14-kDa non-structural protein (NSm). The S segment encodes the nucleoprotein (N) and a virulence factor (NSs). Both viral RNA polymerase and N proteins are necessary for viral RNA replication and transcription [8,9], while the glycoprotein makes up the viral envelope and elicits potent neutralizing antibodies [10].

RVFV was isolated in 1930 in Kenya, with a subsequent major outbreak in South Africa in 1951 [11]. The first outbreak outside of Africa was in the Arabian Peninsula in 2001 [12,13]. Due to the changing world climate and commercialization, RVFV spread towards some new RVFV-free regions has accelerated. Although trade embargos on ruminants and products exported in RVF-endemic countries, this disease can also be spread by an infected person, such as in China [14,15] and Canada [16], where infected people were found. Vaccination is as an effective strategy to prevent viral diseases. Several RVFV vaccines have been used in endemic countries. However, worldwide, there are no licensed human vaccines for preventing RVFV infection and only three veterinary vaccines exist commercially, one formalin-inactivated vaccine and two live attenuated vaccines [17,18].

RVFV is one of the 10 priority pathogens cited by the 2017 World Health Organization (WHO) Blueprint list. Although formalin-inactivated and live attenuated vaccines have been licensed for veterinary use, they still have drawbacks [19,20,21,22]. There are several vaccine platforms for RVF vaccine research that have been reported, including a DNA vaccine [23,24], EHV-1 vector [25], a DNA prime with MVA boost [26], NDV-based vector [27,28] and VLP [29] has been reported. Studies have suggested that humoral immunity is sufficient for preventing RVFV, so a vaccine that can elicit rapid humoral immune responses that neutralize RVFV while being low cost, safe, stable and highly effective is urgently needed (http://www.who.int/blueprint/priority-disaese/en/) [17,30].

Here, we report the use of a rabies virus (RABV)-based vaccine vector as an inactivated dual vaccine for RVFV and RABV. We chose to use RABV as our vector for the RVFV vaccine for the following reasons: there are no pre-existing antibodies to RABV, which would affect the use of the vaccine vector; RABV replicates in the cytoplasm so the viral genome does not integrate into the host genome; there is no danger of regaining virulence after RABV virions are inactivated; and the RABV genome can stably accommodate large foreign genes [31]. This vaccine, named rSRV9-Gn, included a codon-optimized version of the RVFV Gn ectodomain (eGn) and RABV G. This vaccine elicited humoral and cellular responses against RVFV in mice. However, despite eliciting high titres of anti-RVFV IgG antibodies, there was no strong induction of neutralizing antibodies.

## 2. Materials and Methods

### 2.1. Plasmids, Cells and Animals

A recombinant RABV vector, rSRV9, was used in this study, and the RVFV Gn gene (GenBank: DQ380208.1) without its transmembrane domain (TM) or cytodomain tail (CD), which was generated by beginning the RVFV glycoprotein at the 4th available start codon to facilitate optimal glycoprotein expression [32], was cloned between the *P* gene and *M* gene of the RABV vector. The plasmids used included the full-length genome cDNA of rSRV9-eGn and four helper plasmids, PCI-N, PCI-P, PCI-L and PCI-G.

For in vitro assays, BSR and NA cells were obtained from the ATCC and maintained in Dulbecco’s Modified Eagle’s Minimal Essential Medium (DMEM; Gibco, Grand Island, NY, USA) supplemented with 5% or 10% foetal bovine serum (FBS; BI, USA).

For in vivo assays, specific pathogen-free (SPF) female Kunming adult and pregnant mice, which were purchased from the Changchun Yisi Laboratory Animal Technology Co., Ltd. (Changchun, China) and housed individually in standard-size cages, were used as models. Mice were fed standard rodent chow and provided water ad libitum. All experiments requiring injection of RABV were carried out in a special laboratory (BSL-2) designed for in vivo infectious experiments. All of the mice were sacrificed after a certain survival time in accordance with the experimental schedule.

### 2.2. Construction of Full-Length cDNA Clones

Chemically synthesized RVFV Gn (GenBank: DQ380208.1) was amplified with the paired primers RVFV-eGn-F and RVFV-eGn-R (Table 1). Both the linearized vectors and the target gene were amplified using Phusion High-Fidelity DNA Polymerase (New England BioLabs, MA, USA) to avoid mutation. Finally, the target gene was cloned into the BsiWI and PacI sites of rSRV9. The plasmids were verified by PCR amplification and sequencing to ensure correct insertion of the sequence.

### 2.3. Rescue of a Recombinant Virus from cDNA

The recombinant virus was rescued. Briefly, BSR cells pre-seeded in 6-well plates (Corning-Costar, Corning, NY, USA) were transfected with the plasmid containing the recombinant cDNA and with four helper plasmids, PCI-N, PCI-P, PCI-L and PCI-G, using TransIT-LT1 Transfection Reagent (Mirus Bio, Madison, WI, USA) according to the manufacturer’s protocol. The supernatant was collected 3 days after transfection, and the cells were supplemented with fresh medium until 7 days after transfection; this collected virus was defined as the first generation. After 3 generations, the rescued virus was examined by direct immunofluorescence assay (IFA) using FITC-conjugated antibodies against the RABV N protein (Merck Millipore, Billerica, MA, USA) (1:200 in PBST). The recombinant virus was passaged in BSR cells. To evaluate the genetic stability of the recombinant virus, the virus was serially passaged 10 times in BSR cells and evaluated by RT-PCR. The eGn gene was amplified and analysed for mutation.

### 2.4. Immunofluorescence Testing of the Recombinant Virus

BSR cells were seeded in 6-well cell plate and infected with RVFV eGn-expressing RABV or control rSRV9 RABV at a multiplicity of infection (MOI) of 0.1 at 37 °C for 1 h. After removing the inoculum and adding fresh DMEM containing 2% FBS, the cells were incubated at 33 °C for 48 h. At the end of the incubation, the cells were washed with phosphate-buffered saline (PBS), fixed in 80% cold acetone for 2 h at −20 °C, washed with PBS and blocked with PBS containing 5% BSA (Sigma-Aldrich, St. Louis, MN, USA). Then, the cells were incubated with a rabbit polyclonal antibody against RVFV Gn (made by our laboratory) for 1 h at 37 °C. Following washing with PBS and incubation with a FITC-conjugated goat anti-rabbit IgG (Bioss antibodies, Beijing, China) in a solution containing 1% Evans blue (Sigma-Aldrich, St. Louis, MN, USA) diluted in a cassette for 1 h at 37 °C, the cells were washed with PBS containing 0.05% Tween-20 three times and air-dried on a glass slide. Images were captured by using a Zeiss fluorescence microscope (Zeiss, Oberkochen, Germany).

### 2.5. Electron Microscopy

The virus particle integrity of the recombinant virus rSRV9-eGn was examined by transmission electron microscopy (TEM). Briefly, BSR cells were infected with rSRV9-eGn at a MOI of 0.1 for 72 h. The supernatant was collected, and viruses were recovered from cultures by pelleting the cell debris at 1500× *g* for 10 min. A drop of the supernatant was placed onto a copper-coated grid (mesh size 200) at room temperature. The grid was then removed, and the excess liquid was drained off by blotting the edge of the grid with a piece of clean filter paper. The grid was floated on a drop of 2% phosphotungstic acid (PTA) for 2 min and air-dried for a few minutes after the excess PTA was removed as before. The grid was viewed using a HITACHI H-7650 transmission electron microscope.

### 2.6. Inactivation of the Virus and Sucrose Purification

Supernatants containing recombinant virus passaged in BSR cells were spun for 10 min at 10,000× *g* to remove cell debris. The virus suspensions were titrated in NA cells and then inactivated by using betapropiolactone (BPL) (Sigma-Aldrich, St. Louis, MN, USA) added at a 1:3000 dilution and incubated overnight at 4 °C with shaking. The next day, BPL was hydrolysed at 37 °C for 1 h, and the inactivated viruses were examined by cytopathogenicity for BPL and the lack of live recombinant virus by IFA during each of the three passages in NA cells.

Virus precipitation was performed using zinc acetate, and virions were purified by sucrose gradient centrifugation. The cell culture media were inactivated and centrifuged at 3000 rpm for 30 min at 4 °C, and the supernatants were harvested. A volume ratio of 1:50 was added to the zinc acetate solution to adjust the pH to 6.8 at 4 °C for 1 h. Then, the solution was centrifuged at 12,000 rpm for 30 min at 4 °C, the virus was precipitated, the supernatant was discarded, and the virus precipitate was dissolved overnight with a saturated EDTA solution. The concentrated supernatant was then centrifuged for 1.5 h at 22,000 rpm through a 20%, 30%, 40% and 55% sucrose cushion to pellet the virus particles. The virion pellets were resuspended in PBS overnight at 4 °C.

### 2.7. Protein Gel Analysis and Western Blotting

Purified virus particles were denatured in a 5× SDS-PAGE loading buffer (reducing) (Beyotime Biotechnology, Shanghai, China) at 95 °C for 10 min. The purified virions were resolved on a 10% SDS-polyacrylamide gel and stained with Coomassie Blue for 30 min at room temperature for total protein analysis or transferred to a nitrocellulose membrane for Western blot analysis.

To investigate the expression level of the RVFV eGn protein, Western blot analysis was performed. Briefly, purified virions were transferred to a 0.45-μm nitrocellulose membrane (GE Healthcare Life Science, Germany) and blocked in PBS containing 5% non-fat dried milk (blocking buffer) at room temperature for 2 h. After blocking, the nitrocellulose membrane was incubated overnight with a monoclonal mouse anti-RVFV Gn antibody at a dilution of 1:200 in the blocking buffer at 4 °C. After washing, the nitrocellulose membrane was incubated for 1 h with a goat anti-mouse antibody conjugated with horseradish peroxidase (HRP) and diluted 1:10,000 in the blocking buffer at room temperature. The results of SDS-PAGE and Western blot were observed with Gel Image System analysis software, version 4.2 (Tanon, Shanghai, China).

### 2.8. One-Step Growth Curves for the Recombinant Virus

To investigate virus growth, pre-seeded NA cell monolayers were infected with the recombinant virus at a MOI of 0.1 to generate one-step growth curves. Briefly, the NA cells were infected with the recombinant virus for 1 h at 37 °C. The inoculum was removed, and the cells were washed three times using DMEM without FBS. Then, DMEM containing 2% FBS was added. The infected cells were incubated at 33 °C, and the culture medium was harvested every 24 h. The virus was titrated in quadruplicate on NA cells through direct fluorescent antibody assays, and the infected cells were observed under a fluorescence microscope.

### 2.9. Lethality in Suckling and Adult Mice

Suckling mice aged 7 days old and adult mice aged 4–6 weeks old served as sensitive models for comparing relative lethality between rSRV9 and the recombinant virus, as rSRV9 is fatal in suckling mice and non-pathogenic in adult mice after intracerebral (IC) inoculation. The recombinant virus and original virus were titrated to 10^6^ median tissue culture infectious dose (TCID_50_)/50 μL with DMEM, the adult mice were inoculated with 50 μL of serial 10-fold dilutions of the viruses via IC inoculation, and the suckling mice were inoculated with 50 μL of the viruses at 10^5^ TCID_50_/50 μL and serial 10-fold dilutions via IC injection. The mice were monitored and weighed daily. The survival of the mice was monitored daily for 21 days, and the mice that died within the first 4 days due to the shock response were excluded.

### 2.10. Vaccine Preparations

The recombinant virus was inactivated as described above. For vaccine preparation, the immunization dose consisted of 1 × 10^7^ TCID_50_ of inactivated recombinant virus, 20 μg of the adjuvant polyinosinic-polycytidylic acid potassium salt (poly (I:C), Sigma-Aldrich, St. Louis, MN, USA) and 55 μL of ISA 201 VG (Seppic, Paris, France) in sterile PBS. The mixture was emulsified and mixed thoroughly at 31 °C. Then, it stood at 21 °C for 1 h.

### 2.11. Immunization Studies Using the Recombinant RABV Vector in Mice

Six- to eight-week-old female BALB/c mice were purchased from the Changchun Yisi Laboratory Animal Technology Co., Ltd. (Changchun, China). Ten mice were inoculated intramuscularly with 1 × 10^7^ TCID_50_ of inactivated recombinant virus with co-adjuvants (poly (I:C) and ISA 201 VG) and boosted twice at an interval of two weeks with the same amount of virus. Groups of mice treated with the rSRV9 virus or poly (I:C) and ISA 201 VG were used as controls and treated according to the same immunization schedule. Blood samples were collected by retro-orbital puncture immediately prior to the priming immunization and once per week after each booster immunization until two weeks post-immunization.

### 2.12. Generation and Production of Enzyme-Linked Immunosorbent Assay (ELISA) Antigens

The eGn protein was produced by transfecting 293F cells with a plasmid that expressed the secreted eGn with a C-terminal Twin-Strep tag. Purification of the Strep-tagged protein from the supernatant of the transfected cells was performed by using a gravity flow strep-tactin superflow column (IBA Lifescience, Gogentine, Germany) according to the manufacturer’s instructions.

### 2.13. ELISA Analysis of the Immune Response to Immunization

To determine the antibody responses to the eGn protein of RVFV, an indirect ELISA utilizing the purified eGn protein was developed. Briefly, plates were coated with 100 μL of purified eGn protein (2 μg/mL) overnight at 4 °C and blocked with 5% non-fat dry milk in PBST (0.05% Tween 20) at 37 °C for 2 h. Serial dilutions of mouse antisera were allowed to bind for 1 h at 37 °C, and the plates were washed with PBST. Subsequently, the mouse antisera were detected by a goat anti-mouse IgG antibody conjugated with HRP (Bioworld, St. Louis, MN, USA) at a dilution of 1:10,000 in 5% non-fat dry milk in PBST incubated at 37 °C for 1 h. The reaction was detected using a tetramethylbenzidine (TMB) substrate (Sigma-Aldrich, St. Louis, MN, USA) at room temperature. IgG isotypes were also assessed by indirect ELISA, and secondary antibodies specific for IgG1, IgG2a and IgG3 (Southern Biotechnology, Birmingham, AL, USA) were used at varying concentrations determined by optimization.

### 2.14. Virus Neutralization Assay (VNA)

To detect virus-neutralizing antibodies (VNAs) in mouse serum, the serum was heat inactivated at 56 °C for 30 min to ensure complement deactivation.

Serum samples from immunized mice were evaluated for neutralizing antibodies against RVFV eGn by a pseudovirion neutralization assay (PsVNA). The PsVNA was based on a modified vesicular stomatitis virus (VSV) strain (Indiana serotype), in which the G protein of VSV was replaced by the firefly luciferase (Fluc) gene. A RVFV pseudovirus (pRVFV) was prepared as described previously [33] and the neutralization experiment performed by using the pRVFV. Briefly, the pRVFV was incubated with 3-fold serially diluted serum samples (30-fold in the initial dilution) from immunized mice in 96-well plates for 1 h at 37 °C, anti-serum from DNA vaccine immunized guinea pig as positive control, and then Hu7 cells were added, followed by culturing for 24 h. Relative light unit (RLU) values were then measured [34], and the calculated reduction values were compared with these values for control wells, and then the 50% inhibition dilution (ID_50_) values of the serum samples were calculated [33,35,36].

The RABV VNA levels in serum samples were determined for individual mice by using a fluorescent antibody virus neutralization (FAVN) test as described previously [36,37]. The titres of RABV VNAs are expressed in international units (IU/mL) by comparison with a standard serum sample equivalent to 0.5 IU/mL of VNAs based on the WHO standard as a reference.

### 2.15. Ex Vivo T Cell Enzyme-Linked Immunospot (ELISpot) Assays for IFN-γ and IL-4

Two weeks after the third immunization, 3 mice from each group were randomly selected and euthanized. Their spleens were harvested into a tissue culture dish, and each spleen was roughly minced and pressed through a 5-mL syringe. The cells were filtered through a 40-μm filter (BD Falcon 40-μm strainer) and centrifuged at 2000 rpm for 10 min at room temperature. The cells were processed by resuspension in a red blood cell lysis buffer and centrifuged at 2000 rpm for 10 min at room temperature twice. The splenocytes were harvested and washed with RPMI 1640 medium (Gibco, San Diego, CA, USA) containing 10% FBS (Gibco, San Diego, CA, USA). Then, the splenocytes were cultured in RPMI 1640 medium containing 10% FBS and stimulated with or without the purified RVFV eGn protein (10 μg/mL). The purified RVFV eGn protein was expressed by 293F cells and purified with a strep gravity flow strep-actin XT superflow column (IBA Lifescience, Gogentine, Germany). Following an incubation at 37 °C in 5% CO_2_ for 36 h, the frequencies of splenocytes producing IFN-γ or IL-4 were measured using mouse ELISpot kits (Mabtech AB, Stockholm, Sweden) according to the manufacturer’s instructions. Spot-forming cells (SFCs) were enumerated by an automated ELISpot reader (AID ELISPOT reader-iSpot, Germany).

### 2.16. In Vitro Lymphocyte Proliferation

In Vitro lymphocyte proliferation studies used splenocytes from immunized mice. The spleen was treated as in the ELISpot experiment. Splenocyte cultures were seeded in 96-well plates at 100 μL per well (2 × 10^3^ cells) and then stimulated with purified RVFV eGn protein (10 μg/mL) at 37 °C in 5% CO_2_ for 24 h. Lymphocyte proliferation was monitored according to the instructions in the technical manual of the commercial reagent TransDetect Cell Counting kit (CCK) (Transgen biotech, Beijing, China). The CCK solution (10 µL) was directly added to each well, the incubation was continued for 3 h, and then the absorbance was measured at 450 nm.

### 2.17. Cell-Surface Molecule Staining

Two weeks after the third immunization, 3 mice from each group were randomly selected and euthanized. The spleen was treated as in the ELISpot experiment. Viable cells were plated in 6-well dishes (Corning-Costar, Corning, NY, USA) at 5 × 10^6^ cells/mL and stimulated with or without the purified RVFV eGn protein (10 μg/mL) at 37 °C in 5% CO_2_ for 36 h. The cells were then labelled with equal volumes of 1:250 dilutions of anti-CD3 PE-Cy5-conjugated, anti-CD4 FITC-conjugated, anti-CD8 PE-conjugated, anti-CD44 APC-conjugated and anti-CD62 PerCP-Cy5.5-conjugated (BD Biosciences, Franklin, VA, USA) antibodies for 30 min at 4 °C. The labelled cells were analysed with a FACSAria TM Cell Sorter (BD Biosciences, Franklin, VA, USA).

### 2.18. Laboratory Facility and Ethics Statement

The treatment of all mice was in accordance with the welfare and ethical guidance of Chinese laboratory animals (GB 14925-2001). The agreement was approved by the Animal Welfare and Ethics Committee of the Institute of Veterinary Medicine of the Military Academy of Sciences (Laboratory Animal Care and Use Committee Authorization, permit number JSY-DW-2018-02).

## 3. Results

### 3.1. Construction of an Attenuated RABV Vector Expressing the RVFV eGn Glycoprotein

**Figure 1:**
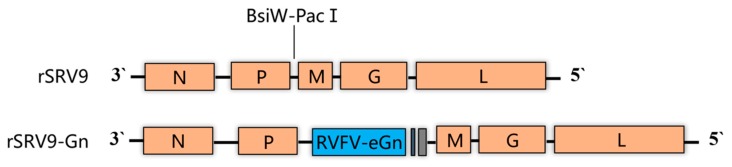
Diagram of the vaccine construct and control construct. rSRV9 is the parental vector, and rSRV9-eGn is derived from rSRV9 with a codon-optimized sequence of the RVFV eGn glycoprotein inserted between P and M via the BsiWI and PacI restriction sites. The blue box represents RVFV eGn (nt 411 to 1746), the grey box represents the transmembrane domain (TM) of the rSRV9 G glycoprotein, and the black box represents the cytoplasmic domain (CD) of the rSRV9 G glycoprotein.

To generate a rSRV9 expressing the RVFV eGn glycoprotein, we used the rSRV9 vector. A codon-optimized RVFV eGn cDNA was cloned into rSRV9 using the BsiWI and PacI restriction sites that flank an rSRV9 transcription start/stop signal between the rSRV9 P and M genes. The rSRV9-eGn construct (Figure 1) expressed a chimeric protein containing the ectodomain of the RVFV M gene C-terminal region (nt 411–1746) and the entire transmembrane domain and cytodomain tail of the rSRV9 G glycoprotein.

**Figure 2:**
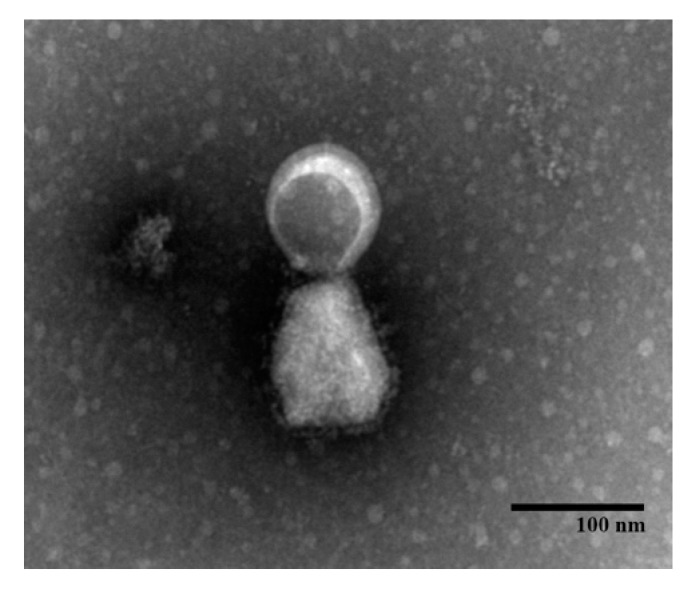
Electron micrograph of the recombinant virus. Recombinant viruses were stained with 1% PTA and analysed by TEM. Scale bar, 100 nm.

To visualize the recombinant virus by TEM, BSR cells were infected with rSRV9-eGn for 72 h, and the recombinant viruses present in precleared culture supernatant were spun for 10 min at 10,000× *g* to remove cell debris and subsequently used to coat carbon-coated Formvar grids. The grids were stained with 1% PTA and analysed by TEM. The recombinant virus retained morphological features of the rhabdovirus, and the size was approximately 100 nm (Figure 2).

### 3.2. RVFV Glycoprotein Expressed by the Recombinant RABV Vector

**Figure 3:**
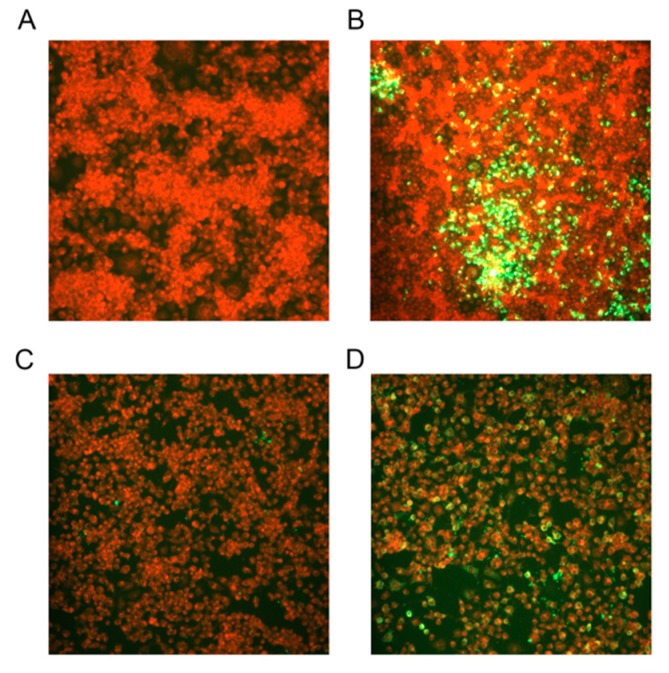
Immunofluorescence experiment. The recombinant virus was successfully rescued, as indicated by direct immunofluorescence, and the recombinant virus was labelled with a mouse anti-RABV N FITC-conjugated antibody. (**A**,**C**) BSR cell controls that were not infected with the recombinant virus lacked fluorescence. Green fluorescence in the cells indicates infection with the recombinant virus (**B**). The RVFV eGn protein in the recombinant virus was labelled with a mouse anti-RVFV Gn monoclonal antibody to identify the successful expression of RVFV eGn from the RABV vector (**D**).

To verify the successful rescue of the recombinant virus and effectively express RVFV eGn, after three generations of blind passaging, a monoclonal antibody against RABV N, which is shown in green, was used to verify whether the recombinant virus rescue was successful (Figure 3A,B). To further verify that the recombinant virus could effectively express RVFV eGn, BSR cells were infected at an MOI of 0.5 with or without rSRV9-eGn for 48 h before immunofluorescence surface staining was performed. Monoclonal antibodies directed against RVFV eGn appear in green (Figure 3C,D).

**Figure 4:**
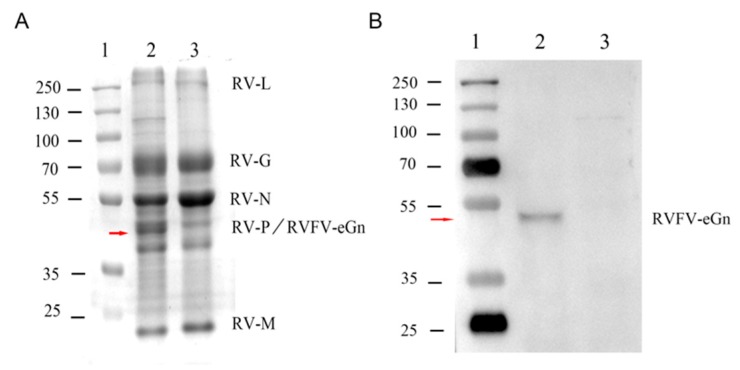
SDS-PAGE and Western blot analysis of virions harvested by sucrose gradient purification. (**A**) The positions of the RABV N, P, M, G, and L proteins were determined by SDS-PAGE. (**B**) Western blot analysis of purified virions probed with a monoclonal antibody against RVFV eGn. In the two images, lane 1 is the molecular mass standards, lane 2 is the rSRV9-eGn, and lane 3 is the rSRV9.

For the inactivated vaccine to induce immunity against both RVFV eGn and RABV G, both glycoproteins must be incorporated into budding virions. To analyse the incorporation of RVFV eGn and RABV G into purified virions, virus particles were isolated from the supernatant of infected BSR cells by filtration and concentration, followed by purification over a 30%–40%–55% sucrose cushion. The virus particles were resolved by SDS-PAGE and Western blotting. The purified rSRV9-eGn virions contained all proteins of RABV and the eGn protein of RVFV (Figure 4A). Since the eGn protein of RVFV is similar in size to the N protein of RABV, eGn was further identified by Western blotting (Figure 4B) by probing with monoclonal antibodies directed against RVFV eGn. eGn proteins migrating at approximately 48.8 kDa were detected. These results further confirmed that RVFV eGn was incorporated into virions. No RVFV eGn protein was detected in purified rSRV9 virions.

### 3.3. Replication and Spread of Viruses with Recombinant Genomes

**Figure 5:**
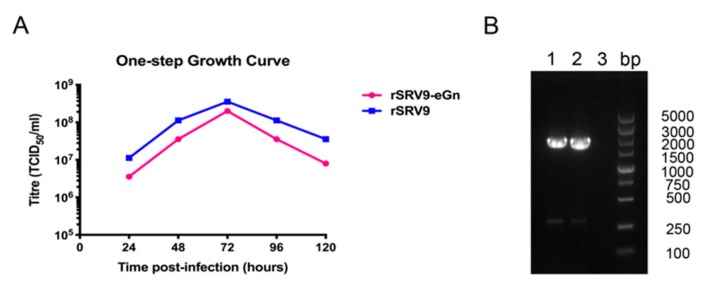
Growth kinetics of recombinant rSRV9-eGn. One-step growth curve comparing the kinetics of live virus replication between rSRV9 (blue) and rSRV9-eGn (pink) in BSR cells. The BSR cells were infected at an MOI of 0.1, and the supernatant was collected at 24, 48, 72, 96 and 120 h. Virus titres were measured by direct immunofluorescence (**A**). Identification of the stability of the insertion of the RVFV eGn gene in the recombinant virus by RT-PCR (**B**).

The growth properties of the recombinant virus were examined in NA cells. The titres of rSRV9 and rSRV9-eGn increased to similar levels at every time point and peaked at 72 h post-inoculation (Figure 5A). In addition, the RVFV eGn gene of the 10th generation recombinant virus was assessed by RT-PCR (Figure 5B) and found to be stably maintained and expressed.

### 3.4. Lethality in Suckling and Adult Mice

**Figure 6:**
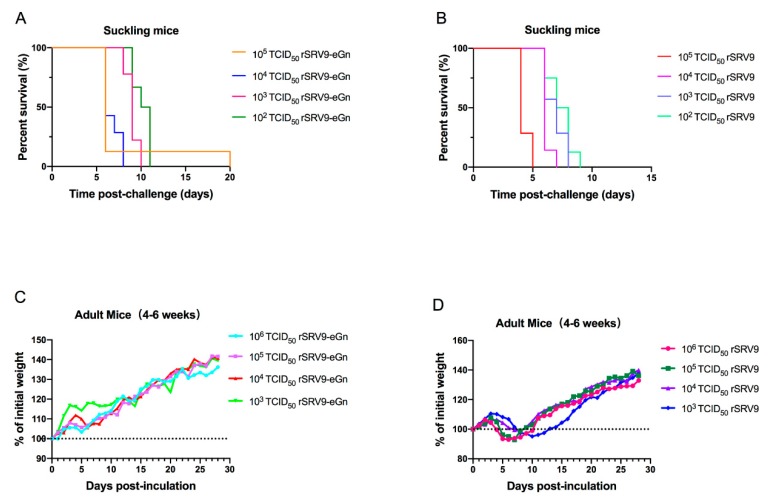
Pathogenicity evaluation of the recombinant rSRV9-eGn virus. Seven-day-old ICR suckling mice were inoculated intracerebrally with 50 μL of solution containing 10^5^ to 10^2^ TCID_50_ recombinant viruses (**A**) or control vector viruses (**B**), and the survival of the suckling mice was monitored daily for 21 days. Groups containing eight 4- to 6-week-old female ICR mice were inoculated intracerebrally with 50 μL of solution containing 10^6^ to 10^3^ TCID_50_ recombinant viruses (**C**) or control vector viruses (**D**), and weight changes were monitored daily for 21 days. Body weight changes in the indicated groups are shown as percentages of the body weight at day 0, which was set as 100.

To investigate whether the expression of RVFV eGn altered the pathogenicity of rSRV9, adult and suckling mice were inoculated intracerebrally with rSRV9 or rSRV9-eGn. Compared with those inoculated with the rSRV9 control virus, the mice receiving rSRV9-eGn experienced significantly delayed lethality; the control mice receiving rSRV9 died between 5 and 9 after injection. In contrast, mice injected with rSRV9-eGn died between 6 to 11 days after injection with the exception of one mouse that survived until day 20. The lethal effects of the rSRV9 control virus and rSRV9-eGn recombinant virus on suckling mice were positively correlated with viral virulence (Figure 6A,B). All of the adult mice inoculated with different viral doses of rSRV9-eGn remained healthy and showed no significant changes in weight, but the adult mice inoculated with different viral doses of rSRV9 showed weight loss within seven days after inoculation (Figure 6C,D).

### 3.5. Humoral Immune Response in Mice

The goal of this study was to develop an improved RABV vaccine against RVFV capable of inducing strong humoral eGn-specific immune responses.

**Figure 7:**
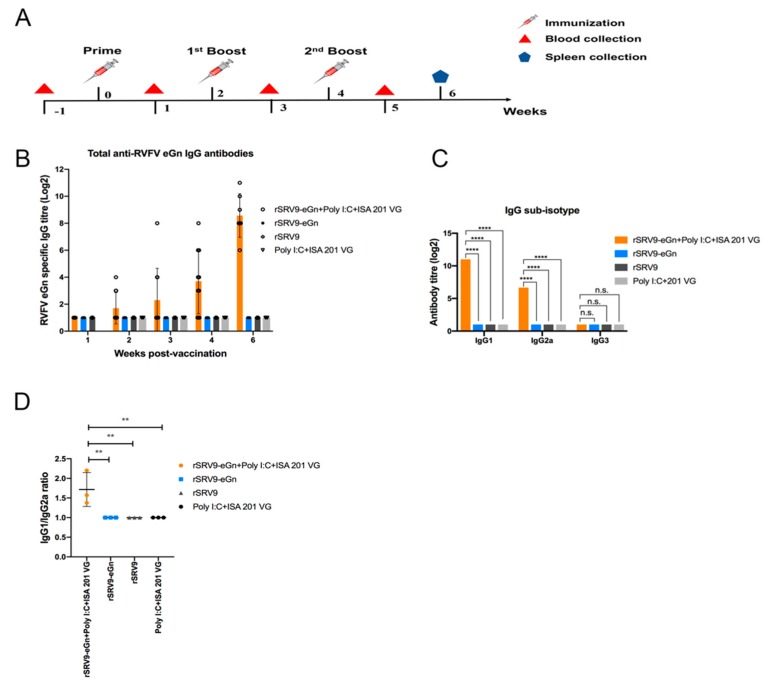
Analysis of the humoral response against RVFV eGn. BALB/c mice were immunized IM in the gastrocnemius muscle with either 10^7^ TCID_50_ of BPL-inactivated virus supernatant with or without poly (I:C) and ISA 201 VG adjuvants or 10^7^ TCID_50_ of rSRV9 vector and boosted two times with the same amount on days 14 and 28 (**A**). ELISA analysis of total IgG against RVFV eGn beginning at one week after the first immunization and continuing until two weeks after the last booster immunization (**B**). Serum antibody titres of IgG subtype (IgG1, IgG2a and IgG3) antibodies against RVFV eGn at two weeks after the last booster immunization (**C**). Data are shown as the mean ± SD and were analysed by two-way ANOVA. The ratios of matched IgG1/IgG2a antibody titres are plotted, and one-way ANOVA was applied to check for variance differences (**D**) (* *P* < 0.05, ** *P* < 0.01, *** *P* < 0.001, and **** *P* < 0.0001).

To assess whether we achieved this goal, we compared serum samples from immunized animals and monitored anti-RVFV eGn antibodies in the serum samples from the mice immunized with inactivated rSRV9, inactivated rSRV9-eGn or inactivated rSRV9-eGn mixed with poly (I:C) and ISA 201 VG (Figure 7B); each group contained 10 mice immunized on days 0, 14, and 28 (Figure 7A).

Blood was collected, and the humoral immune response was analysed weekly until two weeks after the last booster immunization. Analysis of total IgG against RVFV eGn by indirect ELISA indicated seroconversion at two weeks after primary immunization. The mice immunized with inactivated rSRV9-eGn mixed with poly (I:C) and ISA 201 VG produced a specific IgG antibody against RVFV eGn, while the inactivated rSRV9 group showed no anti-RVFV eGn antibody production, indicating that RVFV eGn was expressed from the rSRV9-eGn vector and could stimulate the mice to produce specific IgG antibodies. No specific antibodies were detected in the group immunized with inactivated rSRV9-eGn alone at any of the time points analysed.

We further analysed the isotypes of the induced IgG antibodies against RVFV eGn in the immunized mice. As shown in Figure 7C, IgG1 and IgG2a were the main isotypes detected by IgG subtype detection, and IgG3 antibodies were not detected. The ratios of IgG1/IgG2a indicated the quality of the immune response (Type 1 cellular immune response or Type 2 humoral immune response); ratios > 1 indicate a Type 2 humoral immune response, while ratios < 1 indicate a Type 1 cellular immune response, and a ratio of one indicates a balanced Type 1/Type 2 response. In our study, mice immunized with the rSRV9-eGn vaccine showed a ratio of IgG1/ IgG2a > 1, indicating a Type 2 humoral immune response (Figure 7D).

### 3.6. Antibody Response in Mice

**Figure 8:**
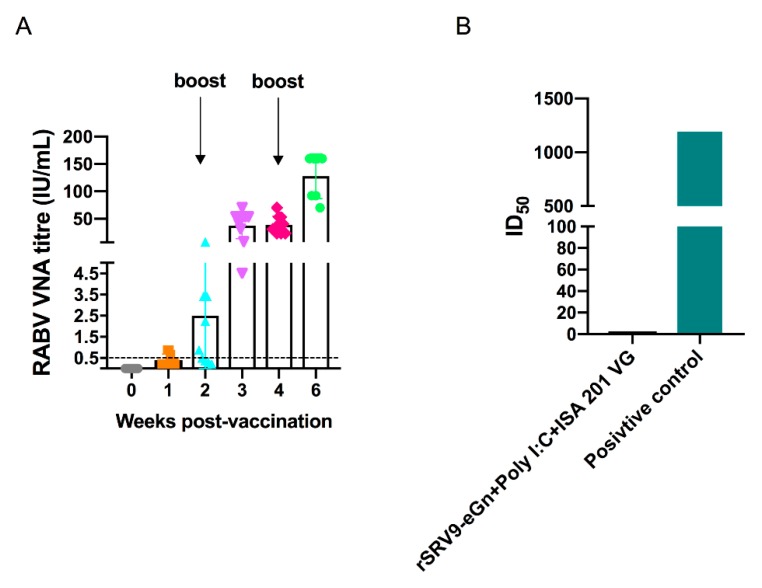
Virus neutralizing antibody (VNA) analysis for immunogenicity evaluation of recombinant rSRV9-eGn in mice. Serum samples collected at two weeks after the last booster immunization were analysed. The titres of VNAs specific for RABV were determined by the FAVN test and are expressed in international units (IU)/mL by using the WHO standard (**A**). The titres of anti-RVFV eGn neutralizing antibodies in immunized mice, compared to those in a positive control serum sample (anti-serum from RVFV DNA vaccine immunized guinea pig) [33], were detected with an in vitro pseudotyped VSV luciferase assay (**B**).

RABV VNAs were detected in the serum of vaccinated mice at different times post-vaccination. One week after primary immunization, 30% (3/10) of the mice were anti-RABV antibody positive, while 70% (7/10) of the mice were anti-RABV antibody positive in the second week; one week after the first booster, all mice expressed anti-RABV antibodies (Figure 8A). A RVFV pseudovirus was prepared using a modified VSV vector, and anti-RVFV eGn neutralizing antibodies were detected using the pseudovirus. Surprisingly, the total IgG titre was highest in the serum harvested two weeks after the last booster immunization, but no anti-RVFV eGn neutralizing antibodies were quantified (Figure 8B).

### 3.7. Vaccine-Induced Antigen-Specific Cellular Immune Response

**Figure 9:**
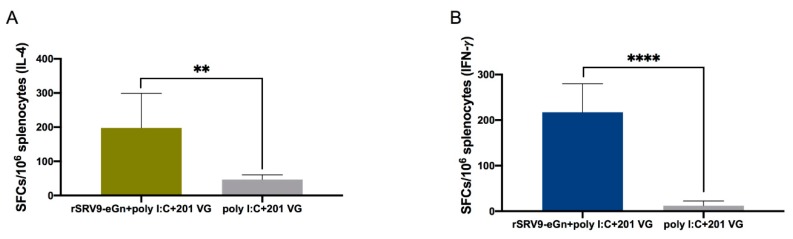
IFN-γ and IL-4 secretion by proliferating splenic T cells induced by a purified RVFV eGn protein. Splenocytes from immunized mice were stimulated with a purified RVFV eGn protein for 36 h, and the levels of IL-4 (**A**) and IFN-γ (**B**) were quantified by ELISpot kits. Data are shown as the mean ± SD and were analysed by an unpaired Student’s test (* *P* < 0.05, ** *P* < 0.01, and **** *P* < 0.0001).

After determining that the recombinant virus could stimulate the production of specific antibodies in mice, antigen-specific cellular immune responses were evaluated by ELISpot assays. Splenocytes were harvested at two weeks after the last booster immunization. As expected, significantly more SFCs for both IFN-γ and IL-4 were detected among the splenocytes from immunized mice (Figure 9A,B) than among those from control mice.

### 3.8. Inactivated Recombinant Viruses Enhance T Cell Proliferative Responses

**Figure 10:**
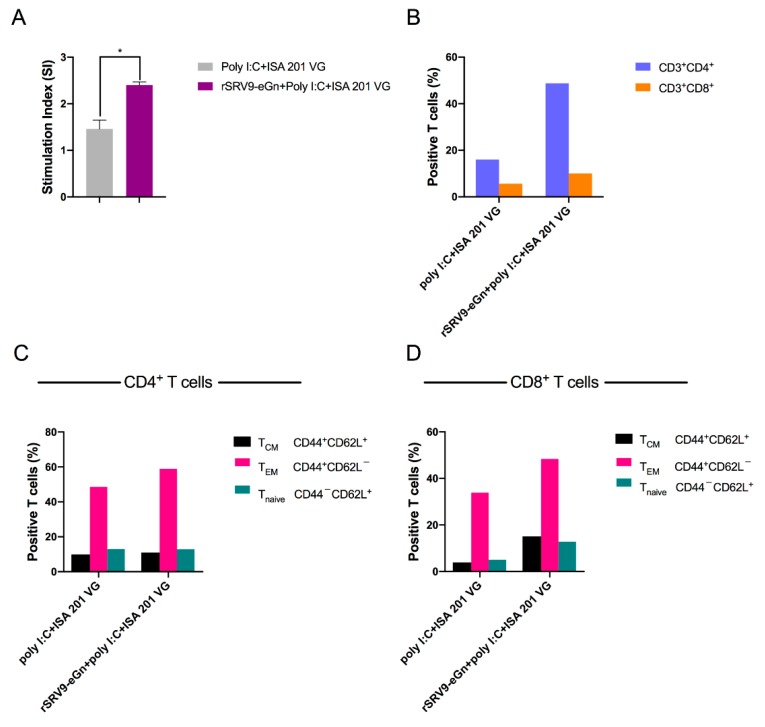
Antigen-specific T cell proliferation. Splenocytes from immunized mice were stimulated with the purified RVFV eGn protein for 36 h. The stimulation index (SI) of the splenocytes was detected using a CCK assay (**A**). T cell proliferation was analysed by using the following gating strategy: live → single cells → CD3^+^ → (**B**) CD4^+^/CD8^+^ → (**C**,**D**) CD44^+^/CD62L^+^. Central memory T cells (T_CM_), effector memory T cells (T_EM_) and naïve T cells (T naïve) were identified using CD44 and CD62L staining.

We investigated the cellular immunity evoked by inactivated recombinant rSRV9-eGn vaccine candidates. First, we examined whether immunization with inactivated recombinant rSRV9-eGn induced antigen-specific T cell proliferation in the spleen. Under stimulation with the purified RVFV eGn protein, the in vitro stimulation index (SI) of the splenocytes of the vaccine-immunized mice was significantly higher than that of the control mice, indicating that the vaccine effectively promoted immune cell proliferation and the immune response (Figure 10A). Using the markers CD44 and CD62L, we discriminated among the following T cell phenotypes: T central memory cells (T_CM_; CD44^+^CD62L^+^), T effector memory cells (T_EM_; CD44^+^CD62L^−^), and naïve T cells (T naïve; CD44^−^CD62L^+^) [38]. The results in Figure 10B, C, and D show that after purified RVFV eGn protein stimulation, the populations of CD4^+^ and CD8^+^ T cells in the vaccine-immunized group increased, and the populations of proliferating CD4^+^ and CD8^+^ T cells were dominated by the T_EM_ phenotype, while the proportion of cells with the T_CM_ phenotype remained low.

## 4. Discussion

RVF outbreaks occur in humans and ruminants. Evidence for the reservoir species for RVFV has pointed towards wild animals and domestic animals. In addition, researchers have investigated whether ruminants, including sheep, goats, and camels, have potential involvement in the occurrence or dissemination of RVF in humans. Furthermore, it is extremely difficult to eliminate the source disease from the reservoir and cut off the transmission from free-range animals through injected vaccines [39]. Therefore, an effective and safe vaccine for livestock against RVFV is of utmost importance and urgency.

The M gene of RVFV encodes a glycoprotein that is cleaved into the Gn and Gc proteins during maturation, and the assembly of the Gn and Gc glycoproteins that surround the RVFV surface constitutes a major target for neutralizing antibody responses during natural infection and immunization [40]. The Gn glycoprotein of RVFV elicits neutralizing antibodies [10,41]. The three subdomains (domains I, II, and III) that comprise the Gn head display different arrangements. Through structural analysis, domain III as been shown to be an ideal region recognized by specific neutralizing antibodies, and domain II is probably also recognized by neutralizing antibodies [42].

In our study, we inserted the ectodomain of Gn, including the head and stem comprising amino acids 154 to 582, into a RABV vector. The recombinant virus was successfully rescued. Compared with the vector virus, the virus with eGn inserted did not show increased pathogenicity. Intracranial inoculation of the recombinant virus into adult and suckling mice resulted in stable weight gain and delayed time of death, respectively (Figure 6). The recombinant virus can be used as a vaccine, safety is ensured by inactivation, and there is no risk of RABV mutation or virulence reversion [31,43].

The data showed that the inactivated vaccine alone had little immunogenicity and needed to be supplemented with an adjuvant. The inactivated rSRV9-eGn vaccine was combined with an aluminium phosphate adjuvant, an aluminium hydroxide adjuvant (data not shown) or poly (I:C) and ISA 201 VG adjuvants, and the results showed that the inactivated vaccine combined with poly (I:C) and ISA 201 VG adjuvants had an improved immune effect. Neutralizing antibodies are considered to be a key factor in protecting against viral infection. In our study, in mice immunized with rSRV9-eGn mixed with poly (I:C) and ISA 201 VG adjuvants, anti-RABV antibodies appeared in the first week after primary immunization, and one week after booster immunization, all mice expressed anti-RABV antibodies (Figure 8A). The results showed that the RABV vector had good immunogenicity. The RVFV eGn gene inserted into the RABV vector was reassembled into a recombinant virus (Figure 2; Figure 3), and RVFV eGn was expressed on the surface of RABV particles (Figure 4A,B). After immunization, mice were found to have RVFV eGn-specific IgG antibodies in the second week after primary immunization (Figure 7B). However, when a modified VSV vector was used to prepare an RVFV pseudovirus for neutralization experiments, surprisingly, the serum from the immunized mice had no neutralizing activity in vitro (Figure 8B). Usually, the natural conformation of an antigen in a vaccine directly affects the immune effect of the antigen. In our study, we inserted the ectodomain of the RVFV Gn protein between the P and M genes of RABV and repackaged a recombinant virus that expressed both the RABV G protein and the RVFV eGn protein. The spatial conformation of the RVFV eGn protein might have affected the production of anti-RVFV eGn neutralizing antibodies after immunization of mice with the vaccine. Inducing animals to produce specific IgG antibodies but not neutralizing antibodies may be related to pathogenic characteristics (such as those of Marburg, Lassa fever, and RVF) and the vector selected for vaccine research. Although no neutralizing antibodies were produced, the vaccine may still provide protection against a challenge with RVFV; other mechanisms such as complement-dependent inhibition, antibody-dependent cellular cytotoxicity (ADCC) and phagocytosis remain to be investigated [23,43,44].

IFN-γ is a Th1-type cytokine involved in the antiviral action of cellular immune responses. IL-4 is mainly produced by Th2 cells and is associated with humoral immune responses. ELISpot results showed that the rSRV9-eGn vaccine could effectively stimulate Th1 and Th2 cytokine production in mice (Figure 9A,B), thereby enhancing cellular and humoral immune responses and regulating the overall immune response. The vaccination regimen applied in our study led to increased levels of CD4^+^ and CD8^+^ T cells (Figure 10A,B). An effective vaccine should establish a long-lived population of memory T cells [45], and the mice in this study generated memory cells among the proliferating T cell population after immunization with rSRV9-eGn, with T_EM_ as the major population (Figure 10C,D).

Differentiating infected animals from vaccinated animals or naturally infected animals during RVF disease outbreaks is of fundamental epidemiological importance. Therefore, differentiation of infected from vaccinated animal (DIVA) compatibility is an important factor to consider when designing vaccines, especially for use in countries or regions nonendemic for RVFV. Using the RVFV glycoproteins and nucleocapsid protein as diagnostic antigens, it may be possible to distinguish vaccine-induced antibody responses. Since rSRV9-eGn contained only RVFV eGn, only RVFV eGn-specific IgG antibodies could be detected, and anti-RVFV N antibodies were not detected in immunized mice. The increase in international trade in livestock coupled with the potential for RVFV outbreaks in nonendemic areas provides strong incentives for the development of DIVA vaccines. The absence of the RVFV nucleoprotein in the vaccine offers the possibility of developing a DIVA vaccine with a companion diagnostic assay using N- and Gn-specific ELISAs [46].

Due to the lack of prophylactic treatment experiments, we cannot predict whether this vaccine can protect animals from RVFV infection since it only induced high titres of RVFV eGn-specific IgG antibodies and no neutralizing antibodies; therefore, efficacy studies still need to be performed. However, this RVFV vaccine designed with RABV as the vector provides ideas for the development of vaccines that prevent RVFV and RABV infections.

## Figures and Tables

**Table 1:** Primers used for construction of the cDNA encoding the MP-12 eGn gene of RVFV.

Primer Name	Sequence (5′-3′)
RVFV-eGn-F	TAT**CGTACG**GCCACCATGGCAGGGATTGCAATGA
RVFV-eGn-R	CC**TTAATTAA**CTAAGTGTGACACTGGTAATTTATCAG

Restriction enzyme sites BsiWI and PacI are shown in bold. The Kozak sequence is underlined.
